# Student perspectives on the use of universal design for learning in virtual formats in higher education

**DOI:** 10.1186/s40561-022-00218-6

**Published:** 2022-12-21

**Authors:** Melissa Beck Wells

**Affiliations:** Department of Educational Studies, State University of New York- Empire State College, 500 Seaview Avenue, Staten Island, NY 10305 USA

**Keywords:** Higher education, Student surveys, Student perspectives, Universal design for learning, Virtual format

## Abstract

Universal design for learning (UDL) in higher education may be a useful tool in supporting the heterogenous higher education student population, specifically in supporting student academic outcomes and retention. With more students enrolling in digital formatted education, specifically international students, a strong framework must be established to support non-geocentric students. Using survey responses of undergraduate degree-seeking students, this mixed-methods study explored the students’ perceptions and impact of UDL in virtual learning modalities (online courses, independent studies, and virtual study groups). A sample size of 57 participants used a Likert-type survey to assess the student-reported prevalence of UDL techniques in higher education in various virtual learning modalities. Students also discussed the strategies that impacted their experience as learners and the mode of study in which they felt most successful. Implications for practice and research are also presented.

Educational equity encompasses the inclusivity of teaching techniques to ensure that diverse learners can access and demonstrate advanced-level knowledge. Higher education students have become are more racially, ethnically, and ability diverse (Espinosa et al., 2019). It is imperative, therefore, that higher education institutions are aware of student needs and have both a plan and guiding framework to ensure all students receive adequate support to achieve the learning institutions’ high standards. To encourage diverse students towards success, a supportive framework must be adopted. According to the National Center for Education Statistics (2021), full-time undergraduate students who had enrolled in a bachelor’s degree program at a four-year degree-granting institution identify as: Black (23%), Hispanics (30%), White (32%), Asians (36%), Pacific Islanders (34%), American Indians/Alaska Natives (27%), and multiracial (25%). In 2020, the employment rate of persons with disability who had a bachelor’s degree or higher was 25.7% in the United States while only 7.6% of people with less than a high school diploma and who had a disability were employed (The Statista Research Department, 2021). The gaps in achievement and issues with retention exemplify that the current higher education systems are not supportive of students and educators in realizing their potential (Hogan & Rose, 2018).

Higher education students, specifically those utilizing online modalities for learning, are increasingly diverse in terms of disabilities, languages, and cultural barriers, and have substantial skill deficiencies. However, higher education instruction has not changed significantly (Lee, 2017) to meet the needs of incoming learners. Inclusive teaching systems must be investigated and systematically implemented to support the changing needs of higher education learners.

In addition to the shift in student demographics, the COVID-19 pandemic and social distancing measures to mitigate its spread have caused changes within higher education institutions. The rapid conversion of many traditional courses to online delivery was unprecedented and conceptualized as emergency online teaching or remote teaching (Hodges et al., 2020). This switch from face-to-face teaching to virtual formats demonstrated the urgent need for flexibility and supportive learning modalities for students and academic staff who may be unfamiliar with these learning formats. As discussed by Hodges et al. (2020), many of the online learning experiences that instructors offer their students will not be fully featured or necessarily well planned, and there is a high probability of suboptimal implementation.

Some students and staff can readily embrace this shift to virtual learning, whereas others require additional support to adapt coursework or to meet the needs of the heterogeneous higher education student population. Walters (2020) highlighted the pressing need to focus on Internet access and investment in the technology needed to close the digital divide in online learning for all students. Rogers-Shaw et al. (2018) argued that, despite the possibilities presented by online education and new technologies, students with disabilities, language barriers, and low socioeconomic status are often less successful in school than students from the non-disabled, English speaking, white males or the dominant culture in higher education (Dolmage, 2018). Further, as discussed by Mohtar and Yunus (2022) “there is a need to learn more about students’ attitudes regarding online learning and how it is utilized in the learning and teaching process.” (p. 203). Consequently, higher education institutions must understand how to best support online learners in a virtual educational environment.

Universal Design for Learning (UDL) provides learning opportunities for all students. Dalton et al. (2019) discussed the core principles of UDL as the creation of varied, accessible, and engaging educational experiences for all students. As stated by Hodges et al. (2020), “…UDL should be part of all discussions around teaching and learning. UDL principles focus on the design of learning environments that are flexible, inclusive, and student-centered to ensure that all students can access and learn from the course materials, activities, and assignments” (p. 7). Planning for UDL, “is a way of responding to changing space and developing technology not with panic and reduction but with planning for hybridity and transformation.” (Dolmage, 2018. p. 131).

The needs of all students should be considered, including students with various learning styles, linguistically diverse students, students who are neurodiverse, and students who benefit from diverse learning strategies. As discussed by Dolmage (2018), “Universal design offers a means of placing those with unconventional abilities, needs, and goals at the center of the design process. When disabled people lead the process, we can more specifically address the power imbalances that lead to exclusive spaces, interfaces and pedagogy.” (p. 129).

By appealing to the heterogeneous diversity in our higher education student populations, the framework of UDL strives to remove discriminatory practices, as the learning needs of most students are considered when instruction is designed. Therefore, it results in the removal of one-size-fits-all teaching practices (Dalton et al., 2019). At the heart of UDL are its three core principles for instructional design: multiple means of engagement, multiple means of representation, and multiple means of action and expression (Rose & Meyer, 2002).

In the 1990s, CAST, Inc. developed the UDL framework for instructional design to guide the inclusive instructional design process, and UDL guidelines to support continued checks for design efficacy. UDL has been increasingly influential in educational systems and policies in the USA, including the Every Student Succeeds Act of 2016 (CAST, 2018). Nelson (2013) mentioned that UDL involves providing deliberately chosen and researched opportunities to all students so they can ultimately understand how to direct their own learning. Creating lifelong learners is the desired outcome.

Although the effectiveness of UDL practices has been discussed, the inclusion of those with learning variabilities has been inconsistent in K-12 settings (De Cesarei, 2015). King-Sears (2014) states that UDL is featured in federal legislation for post-secondary education (Higher Education Opportunity Act of 2008). However, a recent review of UDL research at the post-secondary level yielded scant results. Bracken and Novak (2019) affirm the benefit of integrating a research-based design that establishes a framework ensuring access, engagement, and learning outcomes for all students in higher education institutions. This involves the universal design of post-secondary environments that can meet learning requirements, and the support of students in realizing their learning potentials in “wider worlds of social well-being, creativity, and employment” (Bracken & Novak, p. 3).

Current retention and graduation rates for adult learners demonstrate a need for techniques that support diverse learners. According to Rogers-Shaw et al. (2018), adult educators who follow the well-established but seldom utilized principles of UDL can reimagine how learning occurs and is assessed in the online classroom. It is necessary, therefore, to develop the knowledge of higher education staff in the design of coursework, assessments, and strategies to support diverse learners at higher education institutions.

Post-secondary institutions can support and contribute to one another’s learning knowledge by sharing practices and experiences of using UDL (CAST, 2018). This research seeks to support higher education students by investigating student experiences and perceptions of UDL compliance, as well as the impact of UDL-specific techniques on various virtual learning modalities, and by highlighting in which virtual learning modality students feel the most successful.

## Research questions

This mixed-methods study analyzed students’ perceptions of the prevalence of UDL techniques in higher education in various virtual learning modalities. The research aimed to answer two questions: “What is the student-reported prevalence of UDL techniques in higher education in various virtual learning modes (independent study, online course, virtual study group)?” and “Which mode of learning demonstrates the highest UDL techniques compliance?”

## Methods

A Likert-type survey utilized descriptive statistics to address research question one. The Friedman test and three Wilcoxon signed-rank tests were conducted to address research question two. Furthermore, the responses were triangulated using data from two open-ended questions, which revealed the most impactful mode of study for students and why they chose their preferred study model.

### Participants

A census sampling of undergraduate students at a statewide higher education institution participated in this study. The random sampling protocol focused on enrolled matriculated undergraduate students who had completed at least one course before the current term. Non-matriculated students and students possessing restrictive holds or ‘no survey’ indicators were systematically excluded. All students who did not have these indicators were included in the survey.

### Research instruments

The author created a survey based on the EnACT-PTD (2012) framework, Nine Common Elements of UDL in Higher Education. Each element was developed into an action statement. Students utilized a Likert-type survey to express their perceptions of the frequency of each of the nine elements in the three studied virtual learning modes. The nine elements of UDL were categorized to include strategies that impact the course syllabus, teaching style, teaching resources, student participation in learning, student feedback, and student expression of learning. Students were then asked two open-ended questions to investigate further how to best support learners in higher education. The quantitative and qualitative data collection methods are described as follows.

#### **Procedure**.

An email message was sent to the students’ personal higher education email addresses and included the research procedures. The message also included an email redirect survey invitation link that linked to the survey. Participation was voluntary and anonymous. Participants included students who completed the survey.

#### Data collection: quantitative analytic approach

All analyses were conducted using IBM SPSS v27. First, descriptive statistics were calculated to describe the participants. The descriptive statistics included the overall numbers and percentages of participants in two categories: area of study and number of credits completed. The first research question assessed the reported prevalence of UDL techniques in higher education in various virtual learning modalities and was addressed using descriptive statistics. Total scores for each learning modality (independent study, online course, virtual study group) were calculated, and the minimum, maximum, mean, median, and standard deviation were calculated to determine which UDL techniques in higher education were more prevalent.

The second research question assessed which learning mode demonstrated the highest UDL techniques compliance and was answered using the Friedman test. The participants in the study were treated as one group, and UDL techniques compliance was treated as one measure under three different conditions (independent study, online course, virtual study group). Missing data were handled using listwise deletion because some participants did not respond under all three conditions. The Friedman test results determined whether there was a statistically significant difference between the scores for each condition. However, because there were three different conditions, three post-hoc Wilcoxon-signed rank tests were conducted to determine where the differences occurred: independent study × online course, independent study × virtual study group, and online course × virtual study group. A Bonferroni adjusted alpha value was used to account for the Type 1 error (*p* < .016). Finally, the effect sizes for the differences were calculated by dividing the z value from the Wilcoxon-signed rank test by the square root of the number of observations.

#### Data collection: qualitative analytic approach

Qualitative data analysis was conducted to examine students’ perspectives based on open-ended questions. Students’ perception data were collected to determine which UDL strategies were most impactful to students and the mode of study in which they felt the most successful. Study participants responded to the following questions: (a) Which strategies (if any) do you feel have impacted your experience as a learner? (b) Which mode of study have you felt the most successful in? Why?

To determine and describe the UDL practices within the coursework, an inductive thematic analysis was used. This qualitative analysis allows the UDL instructional practices and patterns of student perspectives to emerge as the data is examined. Thematic analysis in qualitative methods is useful for examining emerging patterns when there is not yet an established body of literature regarding the perceptions of students of UDL techniques in these online learning modes. Thematic analysis is the search for, and extraction of general patterns found in the data through multiple readings of the data.

Inductive thematic analysis was used because the primary goal was for research findings to emerge from the recurrent and prevailing themes in the data. The summary findings derived from the raw open-ended question data are merged to create the meaningful themes and categories demonstrated below, which are relevant to research objectives. The results of inductive analysis are presented through description of the most important themes and categories.

First, the data was cleaned and prepared by editing the text and applying a common format to all data files. Then codes were created through paraphrasing the collected data.

## Results

Study participants were invited to complete a survey about their perspectives on the prevalence of UDL strategies in various learning formats (virtual study group, online course, independent study). The survey questions concerned the Nine Common Elements of Universal Design for Learning in Higher Education (EnACT-PTD, 2012). They also included general open-ended questions regarding the identification of the strategies (if any) that have impacted the students’ experiences as a learner and the identification and rationale of the mode of study in which students felt the most successful.

Of the 57 participants in the study, 30 (53.6%) were in an educational study program, seven (12.5%) were in an interdisciplinary program, five (8.9%) were in a community and human service program, two (3.6%) were in an arts program, and six (10.7%) were undecided. Each of the remaining areas of the study response options had one participant, comprising 1.8% of the sample. In terms of the number of college credits completed, six (10.7%) completed 0–20 credits, nine (16.1%) completed 21–40 credits, seven (12.5%) completed 41–60 credits, six (10.7%) completed 61–80 credits, 12 (21.4%) completed 81–100 credits, and 16 (28.6%) completed 101–124 + credits (Table 1).

**Table 1:** Participant demographics

Variable	Response option	N	%
Area of study	Educational Studies	30	53.6
	Interdisciplinary	7	12.5
	Science, Math, and Technology	1	1.8
	Community and Human Service	5	8.9
	The Arts	2	3.6
	Public Affairs	1	1.8
	B.S. Nursing	1	1.8
	B. S. Accounting	1	1.8
	Business, Management, and Economics	1	1.8
	Cultural Studies	1	1.8
	Undecided	6	10.7
Number of college credits completed	0–20	6	10.7
	21–40	9	16.1
	41–60	7	12.5
	61–80	6	10.7
	81–100	12	21.4
	101–124+	16	28.6

### What is the student-reported prevalence of UDL techniques in higher education in various virtual learning modes (independent study, online course, virtual study group)?

Descriptive statistics were calculated to address research question one, which inquired about the student-reported prevalence of UDL techniques in higher education in various virtual learning modalities. The descriptive statistical analysis results showed that the mean UDL score for the independent study was 30.21 with a minimum score of 17, a maximum score of 36, and a standard deviation of 6.40. Furthermore, the mean UDL score for the online course was 30.52 with a minimum score of 20, a maximum score of 36, and a standard deviation of 5.76. Finally, the mean UDL score for the virtual study group was 32.96 with a minimum score of 25, a maximum score of 36, and a standard deviation of 4.16 (Table 2).

**Table 2:** Descriptive statistics

Descriptives				
			Statistic	Std. error
Independent study total	Mean		30.21	1.19
	95% Confidence interval for mean	Lower bound Upper Bound	27.77 32.64	
	5% trimmed mean		30.58	
	Median		32.00	
	Variance		41.03	
	Std. deviation		6.40	
	Minimum		17.00	
	Maximum		36.00	
	Range		19.00	
	Interquartile range		11.00	
	Skewness		− 0.63	0.43
	Kurtosis		− 1.01	0.84
Online course total	Mean		30.52	1.07
	95% confidence interval for mean	Lower bound Upper bound	28.32 32.71	
	5% trimmed mean		30.78	
	Median		31.00	
	Variance		33.19	
	Std. deviation		5.76	
	Minimum		20.00	
	Maximum		36.00	
	Range		16.00	
	Interquartile range		10.00	
	Skewness		− 0.43	0.43
	Kurtosis		− 1.38	0.84
Virtual study group total	Mean		32.96	0.77
	95% confidence interval for mean	Lower bound Upper bound	31.38 34.55	
	5% Trimmed Mean		33.22	
	Median		36.00	
	Variance		17.32	
	Std. deviation		4.16	
	Minimum		25.00	
	Maximum		36.00	
	Range		11.00	
	Interquartile range		8.00	
	Skewness		− 0.83	0.43
	Kurtosis		− 1.19	0.84

### Which mode of learning demonstrates the highest UDL techniques compliance?

The first step in determining which mode of learning demonstrated the highest UDL techniques compliance was to conduct a Friedman test. It showed that there was a statistically significant difference among UDL compliance across the three leading modalities (independent study, online course, virtual study group *x*^2^ (2, *n* = 29) = 6.31, *p* < .05; see Table 3). Inspection of the median values showed that the virtual study group had the highest UDL compliance (see Table 4).

**Table 3:** Friedman test statistic

N	29
Chi-square	6.31
*df*	2
Asymp. sig.	0.04

**Table 4:** Learning modality percentiles

	N	25th	50th (median)	75th
Independent study	29	25.00	32.00	36.00
Online course	29	26.00	31.00	36.00
Virtual study group	29	28.00	36.00	36.00

Three Wilcoxon signed-rank tests were conducted to determine which learning modalities were significantly different from one another. According to the results, there was no significant difference in UDL compliance between the online course and independent study learning modalities, *z* = − 1.35, *p* = .18, *r* = .14 (see Table 5).

**Table 5:** Wilcoxon signed-rank test statistic, online course-independent study

Z	− 1.354
Asymp. sig. (2-tailed)	0.176

For the virtual study group and independent study learning modalities, the Wilcoxon signed-rank test revealed a statistically significant difference in UDL compliance, *z* = − 2.77, *p* = .006, *r* = .36 (see Table 6).

**Table 6:** Wilcoxon signed-rank test statistic, virtual study group-independent study

Z	− 2.775
Asymp. sig. (2-tailed)	0.006

For the virtual study group and online course learning modalities, the Wilcoxon signed-rank test revealed a statistically significant difference in UDL compliance, *z* = − 2.70, *p* = .007, *r* = .34 (see Table 7). The results of the three Wilcoxon signed-rank tests indicated that a statistically significant difference occurred in the virtual study group.

**Table 7:** Wilcoxon signed-rank test statistic, virtual study group-online course

Z	− 2.696
Asymp. Sig. (2-tailed)	0.007

Responses from the two open-ended questions were analyzed and categorized according to the theme. Participant responses with two-part answers identifying more than one area of difficulty were categorized according to the different themes related to the answers. Because both questions involved two-part responses, percentages were determined according to total responses rather than total participants.

The first question, which asked respondents to identify strategies (if any) that had impacted their experience as a learner had a total of 33 participants and 30 unique responses. Of these responses, 26% revealed that clear feedback from the professor was most impactful, and 26% also indicated that rubrics and clear instructions before the assignment were most impactful. 10% of comments remarked upon the importance of real-life connections to the content, whereas another 10% discussed collaboration techniques with other students. Finally, 13% of the participants commented on time management techniques. There was one unique response related to reinforcing lectures with content materials; three unique responses were blank or stated none (Table 8).

**Table 8:** Thematic coding of open-ended question 1

Thematic category	Percentage (n = 33)	Selected individual responses
Clear feedback	26	If a teacher gives me direct feedback or shows me a specific article Turning around feedback and CLEAR deadlines
Rubrics and clear instructions	26	Teachers give explicit instructions on what should be on a paper. Teacher gives a study guide for a quiz I like when there is an APA template The learning contract helps me clearly provide details of what the professor wants us to have according to the course
Time management	13	Setting aside uninterrupted time to focus on assignments It takes me a long time to read through all the discussion posts in online classes, so I have software that reads to me. It helps a lot Using a planner. I like to put all my course schedules in one place. That way I can see when everything is due in one easy-to-read spot instead of having to look through multiple papers
Real-life connection to content	10	Connecting information to real-world examples I like when a professor can show me an example of something in research and connect it to something I might see every day
Collaboration	10	The use of Moodle was great for me to add more than just my assignments. It also helps me interact with classmates online so that I can use their critique to outline my final papers Break out rooms to engage with other students
Reinforcing lectures	3	Reinforcing lectures with reading materials

The second question, which asked respondents to identify the mode of study in which they felt the most successful, had a total of 45 participants and 40 unique responses. Of these responses, 25% felt the most successful in virtual study groups, while 40% felt the most successful in online courses. 40% of comments remarked about independent studies, while another 7% commented that they felt the most successful in person. Five unique responses were blank or did not mention the mode of the study (Table 9).

**Table 9:** Thematic coding of open-ended question 1

Thematic category	Percentage (n = 40)	Selected individual responses
Virtual study group	25	Virtual Study Group- I save travel time but receive F2F (face-to-face) instruction from the teacher VSG- I can remain at home and be comfortable, but I also get to interact with my peers, ask questions, and get feedback right away. They are funny sometimes too VSG- PowerPoint, video, teacher lecture, small group, and the whole group
Online	40	Online- I only take online courses due to my schedule I only take online courses because of my schedule and because I am comfortable with these courses Well, due to COVID and working remotely for 12 months, I think online works for me because the professor gives you all the materials and assignments and time to submit things. They also explain each step and remind you when things are due
Independent study	40	Independent studies where the professor is a good communicator Independent study b/c I feel like I can go more at my own speed. Plus, I find the discussion post is not open-ended enough. I feel like I’m doing the post and the responses to meet a requirement, not to discuss people’s different views on a topic
In-person	7	In-person. This has helped me be hands-on and not feel as though I must teach myself the lesson to better understand and complete an assignment

## Discussion

The information collected through the descriptive statistical analyses and a Wilcoxon signed-rank test were used to answer the study’s two research questions. Analysis of research question one yielded that the highest mean UDL score was observed for the virtual study group, followed by the online course. The independent study had the lowest mean UDL scores. For research question two, a Friedman test yielded a statistically significant difference among the UDL scores across the three modalities; Wilcoxon signed-rank tests indicated that the mean UDL score for the virtual study group was significantly higher than the scores for the online course and independent study.

The qualitative data indicated that clear feedback, instructions, and rubrics were essential to the learning process of virtual higher education students. Furthermore, instructors who made real-life connections ensured the collaboration of students and reinforced their lectures with appropriate reading material, which was crucial for student success. Lastly, ensuring that students possessed time management skills when completing virtual learning coursework was an important component of student success.

This research has the potential to inform higher educational practices, create more inclusive educational frameworks, and increase student retention and graduation for both traditional and non-traditional students. Strengthening educational practices based on these results will also build equitable and inclusive for all higher education students in online learning environments.

### Implications

The efficient implementation of UDL strategies into all virtual modes may require professional development, access to UDL strategies, college-wide support systems, and experts to mentor faculty in UDL compliance. These UDL elements developed based on the CAST (2018) research represent a starting point for educators in higher education. Incorporating the UDL elements and providing support services for adaptive technology, time management techniques, and access to varied virtual formats were also considered essential to student success. Nevertheless, further empirical research is required to test the effectiveness of such principles in terms of student retention among diverse student populations.

The Nine Common Elements of UDL in Higher Education framework could be useful for further empirical research, especially to understand how these specific strategies impact students within diverse groups (students with disabilities, linguistically diverse students, and students with identified gaps in academic skills when enrolled). Case studies and longitudinal studies may be beneficial for examining the nature of UDL in virtual formats. Moreover, there may be discrete patterns in the balance between teacher training in UDL techniques and student success.

### Conclusion

The present study investigated the prevalence of UDL techniques in virtual formats. The findings have implications for students in higher education and their ongoing efforts to achieve positive learning outcomes. At the organizational level, higher education institutions can best support students by incorporating the teaching strategies rated highest by students, ensuring UDL compliance in all virtual formats, and ensuring students have access to various virtual learning formats. Positive social change will occur by informing new policies that can reduce challenges for higher education students by using sound teaching practices, such as UDL principles, to ensure diversity, equity, and inclusion in graduation rates and in the workforce.

The combination of diverse higher education students and the need for virtual teaching formats has resulted in the present study objective to increase knowledge of the best UDL practices in higher education. It is the responsibility of higher education institutions to obtain knowledge to support diverse learners and create more inclusive environments. Ensuring higher educators have access to this information via professional learning may be one way to disseminate new research findings and ensure equity in teaching practices, as discussed by Bradshaw (2020).

The limitations of this study include the small sample size. This study would benefit from being conducted at several institutions throughout a geographic area, to further demonstrate need across higher education. Also, self-reported data is limited by the fact that it rarely can be independently verified. However, student perspectives were essential to students demonstrating knowledge of and advocacy in UDL strategies which best supported them.

Due to the recent need for more robust virtual learning options and modes, current practitioners are responsible for executing best learning practices of all higher education students, which can be demonstrated by these UDL components. As discussed by Hodges et al. (2020), effective online learning results from careful instructional design and planning using a systematic model. Hodges et al. (2020) considered the design process and showed how the careful consideration of different design decisions impacts the quality of instruction. This research aims to provide information on design decisions that incorporate UDL techniques by directly asking students about the impact.

## Data Availability

The datasets generated during and/or analyzed during the current study are available from the corresponding author on reasonable request.

## References

[CR1] Bracken, S., & Novak, K. (2019). *Transforming higher education through universal design for learning: An international perspective*. Routledge.

[CR2] Bradshaw, D. G. (2020). Examining beliefs and practices of students with hidden disabilities and universal design for learning in institutions of higher education. *Journal of Higher Education Theory and Practice*, *20*(15), 12–20.

[CR20] CAST. (2018). Universal design for learning guidelines version 2.2 [graphic organizer]. Wakefield, MA: Author.

[CR3] De Cesarei, A. (2015). Psychological factors that Foster or deter the Disclosure of disability by University students. *Psychological Reports*, *116*(3), 665–673. 10.2466/15.PR0.116k26w9. https://doi-org.library.esc.edu/. 25933044 10.2466/15.PR0.116k26w9

[CR4] Dalton, E. M., Lyner-Cleophas, M., Ferguson, B. T., & McKenzie, J. (2019). Inclusion, universal design and universal design for learning in higher education: South Africa and the United States. *African journal of disability*, *8*, 519. 10.4102/ajod.v8i0.519. 31392169 10.4102/ajod.v8i0.519 PMC6676777

[CR5] Dolmage. (2018). *Academic Ableism disability and higher education*. Project Muse.

[CR6] EnACT-PTD. (2012). Nine common elements of universal design for learning in higher education. Retrieved from www.udluniverse.com.

[CR7] Espinosa, L. L., Turk, J. M., Taylor, M., & Chessman, H. M. (2019). Race and ethnicity in higher education: A status report. American Council on Education. https://1xfsu31b52d33idlp13twtos-wpengine.netdna-ssl.com/wp-content/uploads/2019/02/Race-and-Ethnicity-in-Higher-Education.pdf.

[CR8] Ferguson, M. K., Dalton, J., E. M., & Lyner-Cleophas, M. (2019). Inclusion, universal design and universal design for learning in higher education: South Africa and the United States. *African Journal of Disability*, *8*(1), 1–7. 10.4102/ajod.v8i0.519. 10.4102/ajod.v8i0.519 PMC6676777 31392169

[CR9] Hodges, C., Moore, S., Lockee, B., Trust, T., & Bond, A. (2020). The difference between emergency remote teaching and online learning. *Educause Review,* 27. https://er.educause.edu/articles/2020/3/the-difference-between-emergency-remote-teaching-and-online-learning.

[CR10] Hogan, N., & Rose, S. (2018). This is what we came here to do: Literacy at the heart of institutional culture change. *Journal of Adolescents and Adult Literacy*, *62*(3), 337–341. 10.1002/jaal.900.

[CR11] King-Sears, P. (2014). Introduction to “learning disability” quarterly special series on universal design for learning: Part one of two. *Learning Disability Quarterly*, *37*(2), 68–70. 10.1177/0731948714528337.

[CR12] Lee, K. (2017). Rethinking the accessibility of online higher education: A historical review. *The Internet and Higher Education*, *33*, 15–23. 10.1016/j.iheduc.2017.01.001.

[CR13] Mohtar, M., & Yunus, Md. (2022). A systematic review of Online Learning during COVID 19: students’ motivation, Task Engagement and Acceptance. *Arab World English Journal*, *2*, 202–215. 10.24093/awej/covid2.13.

[CR14] National Center for Education Statistics (2021, May). Undergraduate retention and graduation rates. https://nces.ed.gov/programs/coe/indicator/ctr.

[CR15] Nelson, L. L. (2013). *Design and deliver: Planning and teaching using universal design for learning*. Brookes Publishing.

[CR16] Rogers-Shaw, C., Carr-Chellman, D. J., & Choi, J. (2018). Universal design for learning: Guidelines for accessible online instruction.* Adult Learning, 29*(1), 20–31. 10.1177/1045159517735530

[CR21] Rose D. H., & Meyer A. (2002).* Teaching every student in the digital age: Universal design for learning*. Alexandria, VA: Association for Supervision and Curriculum Development.

[CR17] Statista Research Department. (2021, March 4). The employment rate of persons with a disability in the United States in 2020, by educational attainment. https://www.statista.com/.

[CR18] Walters. (2020). Inequities in Access to Education: Lessons from the COVID-19 pandemic. *Inequities in access to education: Lessons from the COVID‐19 pandemic*, *36*(8), 8–8. 10.1002/cbl.30483. 10.1002/cbl.30483 PMC7361713 40477007

